# Granulocyte-macrophage colony-stimulating factor (GM-CSF) in subjects with different stages of periodontitis according to the new classification

**DOI:** 10.1590/1678-7757-2021-0423

**Published:** 2022-03-09

**Authors:** Ahu Dikilitas, Fatih Karaaslan, Esra Özge Aydin, Umut Yigit, Abdullah Seckin Ertugrul

**Affiliations:** 1 Usak University Faculty of Dentistry Department of Periodontology Usak Turkey Usak University, Faculty of Dentistry, Department of Periodontology, Usak, Turkey.; 2 IZMIR Katip Celebi University Faculty of Dentistry Department of Periodontology İzmir Turkey IZMIR Katip Celebi University, Faculty of Dentistry, Department of Periodontology, İzmir, Turkey.

**Keywords:** Periodontitis, Gingival crevicular fluid, Interleukins, Macrophages

## Abstract

Granulocyte-macrophage colony-stimulating factor (GM-CSF) is a multifunctional cytokine that regulates inflammatory responses in various autoimmune and inflammatory disorders. Objective: The purpose of this study was to analyze the gingival crevicular fluid (GCF) for GM-CSF, interleukin-1 beta (IL-1β), and macrophage inflammatory protein-1 alpha (MIP-1α) levels in patients with stage I, stage II, stage III, and stage IV periodontitis (SI-P, SII-P, SIII-P, and SIV-P). Methodology: A total of 126 individuals were recruited for this study, including 21 periodontal healthy (PH), 21 gingivitis (G), 21 SI-P, 21 SII-P, 21 SIII-P, and 21 SIV-P patients. Plaque index (PI), gingival index (GI), presence of bleeding on probing (BOP), probing depth (PD), and attachment loss (AL) were used during the clinical periodontal assessment. GCF samples were obtained and analyzed by an enzyme-linked immunosorbent assay (ELISA). Results: GCF GM-CSF, MIP-1α, and IL-1β were significantly higher in SII-P and SIII-P groups than in PH, G, and SI-P groups (p<0.05). There was no significant difference among the PH, G, and SI-P groups in IL-1β, GM-CSF, and MIP-1α levels (p>0.05). Conclusions: These results show that GM-CSF expression was increased in SII-P, SIII-P, and SIV-P. Furthermore, GM-CSF levels may have some potential to discriminate between early and advanced stages of periodontitis.

## Introduction

Periodontitis is a chronic inflammatory disease that is very common in humans.^1^ Periodontitis is characterized by the irreversible destruction of tooth support tissues, and if left untreated, it causes tooth loss, negatively affecting chewing function and aesthetics. Moreover, periodontitis negatively affects systemic health by increasing the risk of systemic diseases such as rheumatoid arthritis and atherosclerosis.^2^ Although periodontitis begins with microorganisms that exist in dental plaque, the severity and progression of the disease are determined by the host’s immune response.^3^ In periodontal disease, the first cells that dominate the inflammatory site are neutrophils, which kill pathogens by means such as phagocytosis and degranulation. During the early stages of periodontal disease, neutrophils arrive at the area of inflammation and kill the pathogens. However, in the later stages of the disease, neutrophils become hyperactive.^4^ Neutrophils that have become hyperactive increase the release of superoxides, pro-inflammatory cytokines, and some destructive enzymes that play a role in tissue destruction.^5^ Neutrophil functions are heavily regulated by colony-stimulating factors (CSFs).^6^

Granulocyte-macrophage colony-stimulating factor (GM-CSF) is a member of the CSF family and is produced from hematopoietic progenitor cells.^7^ GM-CSF plays a critical role in inflammation and autoimmunity and stimulates hematopoietic progenitor cells in the bone marrow to form neutrophils, eosinophils, monocytes, and macrophages. It also promotes the activation and survival of monocytes and macrophages in inflamed tissues.^8^ Clinical findings have shown that GM-CSF is associated with various inflammatory diseases, such as rheumatoid arthritis, atherosclerosis, multiple sclerosis, and lung diseases.^8,9^ An animal study, conducted by Lam and colleagues, showed that experimental periodontitis decreased when GM-CSF had been blocked.^10^ Studies have reported that GM-CSF induces periodontal inflammation in response to periodontal bacteria and plays an important role in pathological bone destruction.^11–13^

Macrophage inflammatory protein-1 alpha (MIP-1*α*) is a biologically active chemokine that helps monocytes and/or osteoclast progenitor cells to become active osteoclasts.^14,15^ Chemokines have been reported to have strong effects in the removal of neutrophils from inflammatory sites.^16^ It has also been found that GM-CSF regulates the expression of pro-inflammatory cytokines, such as tumour necrosis factor (TNF), interleukin 6 (IL-6), interleukin 1β (IL-1β), and chemokine receptors, as well as increasing the migration of neutrophils to peripheral tissues.^8,17^ A study conducted by Zhang and colleagues reported that patients with periodontitis showed a significant increase in the level of chemokines that are related to granulocyte-macrophage colony-stimulating factor (GM-CSF) in gingival tissues. Also, anti-G-CSF antibody therapy importantly decreased the messenger ribonucleic acid (mRNA) release of chemokines, IL-1β, IL-6, and matrix metalloproteinase 9 in periodontal tissues. According to these findings, it is thought that blocking G-CSF results in low neutrophil release from chemokines.^13^

Neutrophils have a very important role in the development of periodontitis. GM-CSF, which has a key role on neutrophil functions, is thought to be effective on the expression of IL-1β and MIP-1*α* in periodontal inflammatory tissues. In previous studies, IL-1β, GM-CSF, and MIP-1*α* changes in gingival crevicular fluid (GCF) were investigated in individuals with periodontitis, but no study has evaluated these biomarkers together at different stages of the disease.^18–20^ The hypothesis of this study is that GCF GM-CSF, which is thought to be effective on the release of IL-1β and MIP-1*α*, may increase with the progression to later stages of periodontitis. Therefore, the purpose of this study was two-fold: first, to conduct comparative research into the GCF GM-CSF, IL-1β, and MIP-1*α* levels of periodontal healthy (PH), gingivitis (G), and periodontitis individuals, and second, to investigate the correlation between biochemical parameters and clinical parameters.

## Methodology

### Patient selection

Between July 2020 and April 2021, 126 participants (61 men and 65 women), aged from 28 to 48 years (mean age = 37.65±4.26 years) were enrolled in a study conducted at the Department of Periodontology, in the Faculty of Dentistry at Uşak University. This study was verified by the Ethics Committee of Usak University’s Faculty of Medicine (protocol number: 80-02-01) and conducted in compliance with the Declaration of Helsinki. This study was recorded on the clinical studies registry NCT04629313. First, individuals were informed of the purpose and design of this study. Then, written informed consent was provided by them.

### Exclusion and inclusion criteria

Individuals’ medical and dental histories were obtained, and those who had systemic diseases, such as diabetes and immunological diseases affecting inflammatory conditions, were excluded from the study. Also excluded from this study were smokers, pregnant women, breastfeeding women, those who had received periodontal treatment in the previous six months, and those who used antibiotics or anti-inflammatory drugs. The subjects included in the study consisted of non-smoking individuals who had no systemic diseases and at least 16 permanent teeth in their mouths.

### Clinical measurement

Plaque index (PI), gingival index (GI), and the presence of bleeding on probing (BOP) were measured by an experienced and calibrated periodontist (AD) who was unaware of the study groups and used a manual probe (Williams, Hu-Friedy, Chicago, IL) in four regions of each tooth, except for the third molars.^21–23^ Measurements for probing depth (PD) and attachment loss (AL) were measured from six regions of each tooth, except for the third molars. According to the information obtained from participants, tooth loss due to periodontitis was assessed.

Calibration exercises were performed in five periodontitis patients who had not been included in the study, and the intra-examiner reliability of the parameters within the study was obtained. The intra-class correlation coefficients for PD and AL were 0.88 and 0.84, respectively.

The study groups were created based on the new classification made in 2017:^24^

The PH group (n=21) included patients with clinically healthy gingivae, BOP < 10%, no attachment and bone loss, and PD lower than 4 mm.

The G group (n=21) included patients with BOP ≥ 10%, no attachment and bone loss, and PD lower than 4 mm.

The Stage I periodontitis (SI-P) group (n=21) included patients with interdental AL = 1–2 mm with PD lower than 5 mm, radiographic bone loss < 15%, and no tooth loss due to periodontal disease.

The Stage II periodontitis (SII-P) group (n=21) included patients with interdental AL = 3–4 mm with PD lower than 6 mm, radiographic bone loss = 15–33% at the coronal third, and no tooth loss due to periodontal disease.

The Stage III periodontitis (SIII-P) group (n=21) included patients with interdental AL ≥ 5 mm with PD greater than 5 mm, radiographic bone loss advancing to the middle or apical part of the root, and tooth loss (< 5) due to periodontal disease.

The Stage IV periodontitis (SIV-P) group (n=21) included patients with interdental AL ≥ 5 mm with PD greater than 5 mm, radiographic bone loss advancing to the middle or apical part of the root, and tooth loss (< 4) with a confounding factor due to periodontal disease.

Patients were excluded from the study if they had AL due to nonperiodontal reasons, such as root caries and gingival recession.^24^

### Measurement of gingival crevicular fluid

First, clinical periodontal measurements were taken; then, 24-48 hours later, GCF samples were taken in the morning using strips of filter paper (e.g., PerioPaper or ProFlow). GCF samples were taken from the interproximal region of the buccal portion of two non-adjacent, single-rooted teeth. For the healthy control group, GCF was taken from areas without inflammation and BOP, whereas for the G group, GCF was taken from areas with inflammation and BOP but without attachment and bone loss. For the periodontitis group, GCF was taken from the regions with the highest PD and bone loss. For the SI-P group, GCF was taken from sites with BOP, PD lower than 5 mm, and radiographic bone loss <15%. For the SII-P group, sites with PD ≤ 5 mm, radiographic bone loss of 15–33% at the coronal third, and BOP were selected. For the SIII-P and SIV-P groups, sites with PD greater than 5 mm, radiographic bone loss advancing to the middle or apical part of the root, and BOP were selected. After plaque was removed from the teeth, the sites were isolated and air-dried. Paper strips were then placed into the pocket until slight resistance was felt, left for 30 s to absorb the GCF and placed in sterile tubes. Samples that were contaminated with oral fluids were excluded from the study. All samples were frozen at −40°C until the time procedures began.

### GCF ELISA method for IL-1β, GM-CSF, and MIP-1*α*

After the paper strips had been placed in Eppendorf centrifuge tubes, 300 ml of phosphate buffered saline at 7.4 pH were added to them. The ingredients in all the tubes were separated using an orbital shaker for 20 min at 240 rpm and centrifuged at 13,000 rpm for 5 min at +4°C. IL-1β, GM-CSF, and MIP-1α levels of the GCF were estimated using enzyme-linked immunosorbent assay (ELISA) kits (Human IL-1β ELISA kit, Human GM-CSF ELISA kit and Human MIP-1α ELISA kit, Elabscience, Texas, USA) according to the manufacturer’s guidelines. Analysis was made in duplicate. Before the samples were added to the wells coated with antibodies specific to IL-1β, GM-CSF, and MIP-1α, the standards in the kits were diluted according to the manufacturer’s instructions. A stop solution was added to each well, and absorbance values were determined by a spectrophotometric ELISA reader (Microplate Reader; Biotek, Winooski) at a wavelength of 450 nm, and then, concentrations were evaluated using standard curves based on absorbance readings. For each plate, the absorbance value was determined according to the concentration of the standards, and the calibration curve was added. The total amount of IL-1β (pg/30s), GM-CSF (ng/30s), and MIP-1α (pg/30s), collected in 30 seconds, was determined and the lowest determining limits were 2.6 pg/30s, 0.4 ng/30s, and 6.3 ng/30s for IL-1β, GM-CSF, and MIP-1α, respectively.

### Statistical analysis

The G*Power 3.1 software package was used to determine adequate examples of the volumes in the study. According to estimates, the sample size that would provide 0.05 type I errors, a 0.89 effect size under one-way ANOVA, and 90% test power was determined to be at least 21 people in each group. To provide power analysis, then, the study continued until data had been collected for 21 patients in each group. The observed power, according to the post-hoc power calculation, was 89%.

Shapiro-Wilk normality tests were used to determine whether variables adapted to normal distribution or not. Considering that data were abnormally distributed in the comparison between groups, the non-parametric Kruskal-Wallis test, followed by the Dunn-Bonferroni post hoc test, and frequency data were evaluated using the chi-square test. The correlation between clinical parameters and the IL-1β, GM-CSF, and MIP-1α levels in the GCF was evaluated using Spearman’s rank correlation analysis. Covariance analysis was used to examine the relationship between different periodontal conditions (healthy/low AL group versus high/severe AL group) and biomarker levels after adjusting for age, sex, and number of teeth. Odds ratio (OR) of the independent variables to the periodontal disease groups were evaluated by logistic regression. Data were analyzed using the statistical software program SPSS (v. 22.0, IBM), and p<0.05 was considered statistically significant.

## Results

### Clinical parameters

Table 1 shows the demographic data of the study groups. Mean age was significantly higher in the SIV-P patients than in the PH, G, SI-P, SII-P, and SIII-P groups (p<0.05). There were no significant differences in age and number of teeth between the study groups (p>0.05).

**Table 1:** The demographic characteristics of the study groups

Demographic variables	PH	G	SI-P	SII-P	SIII-P	SIV-P
	(n = 21)	(n = 21)	(n = 21)	(n = 21)	(n = 21)	(n = 21)
Age (years)	36 (30-48)	39 (30-40)	38 (30-41)	36 (30-44)	38 (28-42)	40 (36-48) *
Gender (female/male)	11 10	12 9	10 11	11 10	11 10	10 11
Number of teeth	27 (24-28)	26 (24-28)	26 (25-28)	26 (24-28)	25 (24-26)	25 (23-27)

Abbrevations: PH, periodontally healthy; G, gingivitis; SI-P, SII-P, SIII-P, and SIV-P, stage I, stage II, stage III, and stage IV periodontitis; Kruskal-Wallis test All data (except gender) are given as median (min-max)

* p<0.05

When clinical periodontal parameters were evaluated; full-mouth and sampling site PD, AL, and PI clinical parameters in the periodontitis groups were significantly higher than those in the PH and G groups (p<0.05). Full-mouth and sampling site GI and BOP parameters in the periodontitis groups were also significantly higher than those in the PH group (p<0.05) (Table 2-Table 3).

**Table 2:** Clinical periodontal parameters and GCF total amount of biomarkers in the study groups

		PH	G	SI-P	SII- P	SIII- P	SIV- P
		(n=21)	(n=21)	(n=21)	(n=21)	(n=21)	(n=21)
Full mouth	PD (mm)	1.8 (1.3-2.3)	2.2 (2-2.8)	4 (4-4.2)	4.1 (4-4.4)	6.5 (6.-6.8)	6.8 (6-7)
	AL (mm)	0 (0-0)	0 (0-0)	2 (1.5-2.5)	3 (2.5-4)	5 (5-5.8)	5.5 (5.2-6)
	GI	0.12 (0-0.4)	2 (1.6-2.2)	2 (1.6-2.2)	2.2 (1.2-3.1)	2.1 (1.2-3.1)	2.4 (2-2.8)
	BOP (%)	1.7 (1-2)	78.5 (58.2-88)	78 (71.6-80.2)	82.8 (78.6-88)	80 (71.6-88)	84.7 (80-86.7)
	PI	0.3 (0-0.7)	1.5 (1-2)	2.1 (1.6-2.9)	2.9 (2.1-3.3)	3 (2.2-3.2)	3 (2.9-3.2)
Sampling site	PD (mm)	2 (1.7-2.3)	3 (3-3)	4.4 (4-4.9)	5 (4.6-5.4)	6.7 (6-7.5)	7 (6-7.2)
	AL (mm)	0 (0-0)	0 (0-0)	2 (1.5-2.8)	3.5 (2.5-4)	5.5 (5-6)	5.8 (5.5-6)
	GI	0 (0-0)	2.2 (1.9-2.3)	2.3 (1.9-2.3)	2.3 (2.1-3.1)	2.3 (1.9-3.1)	2.5 (2.2-3.1)
	BOP (%)	0 (0-0)	100 (100-100)	100 (100-100)	100 (100-100)	100 (100-100)	100 (100-100)
	PI	0.2 (0.1-0.6)	2 (1-2.8)	2.6 (1.6-3.4)	3 (2.6-3.4)	3 (2.3-3.2)	3.2 (2.9-3.3)
Total amounts of biomarkers	GM-CSF (ng/30s)	0.59 (0.4-1)	0.62 (0.4-1.8)	0.58 (0.4-1.8)	1.15 (0.6-4.9)	1.55 (0.6-5.3)	1.02 (0.5-1.9)
	IL-1β (pg/30s)	4.07 (2.7-6.8)	3.94 (2.6-13.9)	5 (2.6-10.9)	14.31 (6.7-45.4)	15.09 (2.7-115)	36.78 (8.7-52.7)
	MIP-1α (pg/30s)	8.44 (6.3-11.8)	8.98 (6.3-18.3)	9.82 (7.2-12.6)	11.65 (6.5-27.9)	16.49 (6.5-21.3)	13.21 (8.3-28.2)

Abbrevations: PH: periodontally healthy; G: gingivitis; SI-P, SII-P, SIII-P, and SIV-P: stage I, stage II, stage III, and stage IV of periodontitis; PI: plaque index; GI: gingival index; BOP: bleeding on probing; PD: probing depth; AL: attachment loss; IL-1β: interleukin-1 beta; GM-CSF: granulocyte colony-stimulating factor; MIP-1 α: macrophage inflammatory protein-1 alpha. Kruskal-Wallis test All data are given as median (min-max)

**Table 3:** Bonferroni post hoc test between study groups with clinical parameters and total amounts of biomarker

	Full mouth					Sampling site					Total amounts of bimarkers		
	PI	GI	BOP	PD	AL	PI	GI	BOP	PD	AL	GM-CSF	IL-1β	MIP-1α
PH-G	0.001*	0.001*	0.001*	0.001*		0.001*	0.001*	0.001*	0.001*				
PH-SI	0.001*	0.001*	0.001*	0.001*	0.001*	0.001*	0.001*	0.001*	0.001*	0.001*			
PH-SII	0.001*	0.001*	0.001*	0.001*	0.001*	0.001*	0.001*	0.001*	0.001*	0.001*	0.001*	0.001*	0.001*
PH-SIII	0.001*	0.001*	0.001*	0.001*	0.001*	0.001*	0.001*	0.001*	0.001*	0.001*	0.001*	0.001*	0.001*
PH-SIV	0.001*	0.001*	0.001*	0.001*	0.001*	0.001*	0.001*	0.001*	0.001*	0.001*		0.001*	0.001*
G-SI	0.001*			0.001*	0.001*	0.003*			0.001*	0.001*			
G-SII	0.001*		0.002*	0.001*	0.001*	0.001*	0.001*		0.001*	0.001*	0.001*	0.001*	0.001*
G-SIII	0.001*		0.004*	0.001*	0.001*	0.001*			0.001*	0.001*	0.001*	0.001*	0.001*
G-SIV	0.001*	0.001*	0.002*	0.001*	0.001*	0.001*	0.001*		0.001*	0.001*		0.001*	0.001*
SI-SII	0.001*		0.001*		0.001*	0.001*	0.005*		0.001*	0.001*	0.001*	0.001*	0.001*
SI-SIII	0.001*		0.004*	0.001*	0.001*	0.002*			0.001*	0.001*	0.001*	0.001*	0.001*
SI-SIV	0.001*	0.001*	0.001*	0.001*	0.001*	0.001*	0.001*		0.001*	0.001*		0.001*	0.001*
SII-SIII				0.001	0.001*				0.001*	0.001*			
SII-SIV	0,001*	0,001*		0,001*	0,001*		0,001*		0,001*	0,001*			
SIII-SIV		0,001*			0,001*		0,001*			0,001*			

Abbrevations: PH, periodontally healthy; G, gingivitis; SI-P, SII-P, SIII-P, and SIV-P, stage I, stage II, stage III, and stage IV periodontitis; PI, plaque index; GI, gingival index; BOP, bleeding on probing; PD, probing depth; AL, attachment loss; IL-1β, interleukin-1 beta; GM-CSF, granulocyte colony stimulating factor; MIP-1 α, macrophage inflammatory protein-1 alpha. Kruskal-Wallis/Dun-Bonferroni post hoc test.

* p<0.05

### Biochemical findings

Table 2 shows the total amount of biomarker levels (IL-1β, GM-CSF, and MIP-1α in the GCF). GM-CSF levels were significantly higher in SII-P and SIII-P groups compared to PH, G, and SI-P groups (p<0.05, Table 3). MIP-1α and IL-1β levels were similar in the SII-P, SIII-P, and SIV-P groups and significantly higher than those in the PH, G, and SI-P groups (p<0.05, Table 3).

### Correlations

Positive correlations were also found among full-mouth and sampling site PI, GI, BOP, PD, and AL parameters and the total amount of IL-1β, GM-CSF, and MIP-1α in the GCF (p<0.001 for all, Table 4). Table 5 shows the mean biomarker levels before and after adjustment for the effects of age, sex, and number of teeth between those who had different severities of AL (healthy/low versus high/severe). Differences were significant. Biomarker levels were found to be significantly lower in the group with healthy/low AL, from 0 to 2 mm (p<0.05).

**Table 4:** Correlations between total GCF IL–1β, GM–CSF, and MIP–1α levels with clinical parameters of study groups

Clinical parameters		GM-CSF (ng/30s)	IL-1β (pg/30s)	MIP-1α (pg/30s)
Whole mouth				
	PI	0.394**	0.450**	0.437**
	GI	0.244**	0.355**	0.287**
	BOP	0.278**	0.280**	0.326**
	PD	0.380**	0.553**	0.472**
	AL	0.411**	0.563**	0.519**
Sampling site				
	PI	0.350**	0.414**	0.397**
	GI	0.251**	0.342**	0.351**
	BOP	0.245**	0.263**	0.293**
	PD	0.405**	0.561**	0.511**
	AL	0.422**	0.558**	0.504**

Abbrevations: PI: plaque index; GI: gingival index; BOP: bleeding on probing; PD: probing depth; AL: attachment loss; IL-1β: interleukin-1 beta; GM-CSF: granulocyte colony-stimulating factor; MIP-1 α: macrophage inflammatory protein-1 alpha

**Table 5:** Unadjusted and adjusted scores of biomarker levels by severity of clinical attachment loss

Biomarker	Clinical attachment loss^a^				Statistics	
levels	Unadjusted Scores		Adjusted Scores			
	Healthy/Low	High/Severe	Healthy/Low	High/Severe	F^b^	p
	(n = 64)	(n = 62)	(n = 64)	(n = 62)		
	mean±SD	mean±SD	mean±SD	mean±SD		
GCSFR	0.66±0.28	1.63±1.16	0.53±0.12	0.76±0.12	48.635	0.001*
IL-1B	5.07±2.56	25.53±21.77	5.95±2.1	24.65±2.1	33.746	0.001*
MIP1-ALFA	9.11±2.04	14.59±6.14	9.17±0.6	14.53±0.6	33.712	0.001*

Abbrevations: IL-1β: interleukin-1 beta; GM-CSF: granulocyte colony-stimulating factor; MIP-1 α: macrophage inflammatory protein-1 alpha.

Healthy/Low groups: PH/G/SI-P; High/Severe groups; SII-P/SIII-P/SIV-P

a AL categories (mean full mounth AL): healthy/low: 0 to 2 mm; high/severe > 3.0 mm [37]

b Adjusted for age, gender, and number of teeth; ANCOVA

* p<0.05

Patients were divided into 2 groups: group 1 (PH, G, and SI-P) and group 2 (SII-P, SIII-P, and SIV-P). Some independent variables, such as number of teeth, PI, GM-CSF, and MIP-1α were determined, and a logistic regression analysis examined the effects of these variables on the groups. The effect of independent variables on the model is statistically significant. Most prominently, the probability of finding GM-CSF in Group 2 is 56.83 times greater than in Group 1 (p<0.05, Table 6).

**Table 6:** Results of logistic regression analysis on age, number of teeth, PI, GM-CSF, and MIP-1α levels

	Univariable OR (95%Cl)*	p-value
Age	1.17 (0.74-1.85)	0.497
Number of teeth	0.15 (0.04-0.6)	0.007*
PI (sampling site)	29.54 (2.05- 425.16)	0.013*
GM-CSF	56.83 (4.03-800.32)	0.003*
MIP-1α	1.57 (1.09-2.27)	0.015*

Abbrevations: GM-CSF: granulocyte colony-stimulating factor; MIP-1 α: macrophage inflammatory protein-1 alpha; PI: plaque index Dependent variables: PH, G and SI-P (group 1) and SII-P, SIII-P, and SIV-P (group 2) independent variables: number of teeth, PI, GM-CSF, and MIP-1α; logistic regression analysis.

* p<0.05

## Discussion

Periodontal diseases were reclassified as periodontal and peri-implant diseases and conditions at the 2017 World Workshop. Using this new classification, a fresh perspective has been applied, and periodontitis has been classified by stage and grade. However, current diagnostic approaches, including clinical periodontal measurements and radiographic findings, are insufficient for showing disease activity and the risk of future progression. Periodontitis, a disease that progresses silently, can only be defined in its late stages. Specific biomarkers that would be effective in determining the stage and grade of periodontitis have been noted with question marks in the new classifications.^24^ To make an early diagnosis, it is of great clinical importance to determine the markers that cause the destruction of periodontal tissue in the early period of the disease. In this study, we investigated the GCF GM-CSF, MIP-1α, and IL-1β levels of individuals with PH, G, SI-P, SII-P, SIII-P, and SIV-P according to the new classifications. IL-1β is mainly a member of the IL family, produced by macrophages and commonly found in periodontal tissues.^18^ MIP-1α is an acidic protein secreted by inflammatory cells at sites of inflammation. Moreover, MIP-1α release was associated with the formation and activation of cells that cause bone resorption.^14,15^ GM-CSF is a growth factor produced by hematopoietic progenitor cells. Studies have shown that GM-CSF is associated with various diseases characterized by inflammation, especially rheumatoid arthritis. It is also one of the cytokines found in inflamed joint fluid.^9,25^ Due to the similarity of the inflammatory processes between periodontitis and rheumatoid arthritis, we hypothesized that GM-CSF may play an important role in the pathogenesis of periodontitis. To our knowledge, this is the first study to analyze the GCF, GM-CSF, MIP-1α, and IL-1β levels of patients according to the new classification criteria.

Our study found that GCF GM-CSF and its associated MIP-1α and IL-1β levels increase with the progression to later stages of periodontitis. These increased cytokine levels in patients with periodontitis were found to be significantly lower in individuals who are periodontal healthy. Moreover, strong correlations were found between GCF GM-CSF and clinical parameters for PI, GI, BOP, PD, and AL. The available data support the view that GM-CSF, which plays a role in the pathogenesis of various inflammatory diseases, may be an important factor in determining the severity of inflammatory conditions such as periodontitis.

Potential markers for monitoring periodontal diseases can be studied using different biological fluids, such as saliva, serum, and GCF. GCF is a region-specific fluid consisting of blood, host factors, and a plaque system originating from the plexus of blood vessels under the epithelium.^26,27^ In this study, GCF was used to determine marker levels and, according to our findings, IL-1β, GM-CSF, and MIP-1α levels in the GCF have a good ability to indicate advanced stages of periodontitis. In this study, data were calculated as totals rather than concentrations since these are more reliable and convenient methods for determining periodontal disease markers in GCF.^26^

GM-CSF is a multifunctional cytokine which has a wide spectrum of biological activity in both innate and adaptive immune responses.^8^ Studies have reported that there are high levels of GM-CSF in the synovial fluids and blood-brain barriers of inflammatory diseases, such as rheumatoid arthritis, multiple sclerosis, and arthrosclerosis.^25,28-30^ Although the level of GM-CSF has been studied in many inflammatory fluids, there are few studies that have extensively investigated the level of GM-CSF in GCF. A study by Zhang and colleagues showed that endogenous GM-CSF inhibition in periodontal tissues significantly reduces the severity of periodontitis. Moreover, a GM-CSF blockade has been reported to cause decreased neutrophil accumulation in the gingiva.^13^ Similarly, a study conducted by Kim and colleagues found activated GM-CSF in the gingival tissues of patients with periodontitis.^31^ In a study by Thunell, et al.^32^ (2010), no significant distinction was found between the GM-CSF levels in the GCFs of individuals with periodontitis and those who had good PH. According to our results, GM-CSF levels in the GCF, which correlated positively to the clinical parameters for PD, were found to be highest in individuals with SIII-P, but there were no significant differences in individuals with SII-P, SIII-P, and SIV-P. At the same time, according to the old classification, the group of patients with aggressive periodontitis was considered a match for those with stage III and grade C periodontitis in the new classification. In a study conducted by Teles and colleagues, individuals with aggressive periodontitis had high levels of GM-CSF compared to individuals with good PH.^20^ A study conducted by Oliveira and colleagues found that GM-CSF was significantly higher in aggressive periodontitis, decreasing after periodontal treatment.^33^ Similar to these studies, our study found GM-CSF to be high in individuals with SIII-P. These findings point to the role of GM-CSF in periodontal inflammation, as shown in previous studies.^8^ Moreover, this hypothesis supports the view that GM-CSF increases bone loss, one of the differential consequences of periodontitis.^34^ Tissue destruction in periodontitis indicates insufficient resolution of inflammation as a result of immune cell dysfunctions, which may be another potential comment for the role of this growth factor in periodontal diseases.^35^

One of the hypotheses in our study was that GM-CSF may have an effect on the release of IL-1β and MIP-1α in periodontal inflammatory tissues. GM-CSF has been shown to activate monocytes and macrophages in inflamed tissues, as in rheumatoid arthritis, and activated macrophages affect the release of chemokines, such as IL-1β and MIP-1α.^8^ It has been shown that MIP-1α and IL-1β have important pathological functions in the development of various inflammatory and autoimmune diseases and are at high levels in the regions in which periodontitis is active.^36,37^ Therefore, the current findings of IL-1β and MIP-1α are unsurprising.

As expected, we found that the levels of these pro-inflammatory cytokines were significantly increased in individuals with SII-P, SIII-P, and SIV-P (high/severe AL group) compared to those with good PH, G, and SI-P (healthy/low AL group). Biomarker levels were found to be associated with the severity of AL after adjusting for age, sex, and number of teeth. According to the regression analysis performed in this study, patients with high GM-CSF and MIP-1α levels are more likely to be in the SII-P, SIII-P, and SIV-P groups. Although there are some differences, our results agree with previous studies. We also observed that these pro-inflammatory cytokine levels were similar in patients with G and SI-P. SI-P is the transition stage to periodontitis, in which the first AL is observed. Because G and SI-P are initial, intertwined stages of periodontal disease, cytokine levels are thought to be similar. Moreover, since both biomarker levels correlate well with the clinical indicators of periodontitis severity levels, all these results confirm that patients with periodontitis are in a measurable inflammatory state. All these observations lead us to predict that GM-CSF, which plays an important role in inflammatory diseases, works together with MIP-1α and IL-1β in the pathogenesis of periodontitis. From both a clinical perspective and our results, GM-CSF may be a promising biomarker in identifying advanced stages of periodontitis (SII-P, SIII-P, and SIV-P) and distinguishing them from SI-P.

This study has some limitations. One of its main limitations is that the Periotron device was not used to calculate GCF volume. GCF collected on paper strips consists of residual fluid and inflammatory exudate present in the gingival sulcus. It has been reported that a high GCF volume reduces its concentration as a result of increased local inflammation in diseased areas.^38–40^ Regarding the relation between GCF components and periodontal diseases, it has been reported that the total amount of biomarker may be a more valid and reliable indicator for diagnostic purposes than the concentration.^38–40^ Therefore, for this study, the amount of GM-CSF, IL-1β, and MIP-1α in the GCF was estimated as their total amount, rather than assessing its concentration. Another limitation may be the relatively small sample size of our study, although it is sufficient, according to power estimates. Moreover, serum and saliva samples were unused. Finally, the rate of progression of the disease was not evaluated.

## Conclusion

GM-CSF appears to be an important marker that is expressed in patients with SII-P, SIII-P, and SIV-P. The release of GM-CSF may be a result of the inflammatory state of the local inflammation in SII-P, SIII-P, and SIV-P. Prospective and more extensive research is needed to validate our findings and to understand the impact of this biomarker on periodontal disease.
